# Platelet biomarkers identifying mild cognitive impairment in type 2 diabetes patients

**DOI:** 10.1111/acel.13469

**Published:** 2021-09-16

**Authors:** Haitao Yu, Yanchao Liu, Ting He, Yao Zhang, Jiahua He, Mengzhu Li, Bijun Jiang, Yang Gao, Chongyang Chen, Dan Ke, Jianjun Liu, Benrong He, Xifei Yang, Jian‐Zhi Wang

**Affiliations:** ^1^ Department of Pathophysiology Key Laboratory of Ministry of Education for Neurological Disorders School of Basic Medicine Tongji Medical College Huazhong University of Science and Technology Wuhan China; ^2^ Key Laboratory of Modern Toxicology of Shenzhen Shenzhen Center for Disease Control and Prevention Shenzhen China; ^3^ Department of Neurosurgery Tongji Hospital Tongji Medical College Huazhong University of Science and Technology Wuhan China; ^4^ Key Laboratory of Ministry of Education for Neurological Disorders Li Yuan Hospital Tongji Medical College Huazhong University of Science and Technology Wuhan China; ^5^ School of Physics Huazhong University of Science and Technology Wuhan Hubei China; ^6^ Department of Neurosurgery Wuhan Central Hospital Affiliated to Tongji Medical College Huazhong University of Science and Technology Wuhan China; ^7^ Department of Physiology School of Basic Medicine Tongji Medical College Huazhong University of Science and Technology Wuhan Hubei China; ^8^ Co‐innovation Center of Neuroregeneration Nantong University Nantong China

**Keywords:** Alzheimer's disease, mild cognitive impairment, optineurin, peripheral biomarkers, platelet, proteomics, type 2 diabetes mellitus

## Abstract

Type 2 diabetes mellitus (T2DM) is an independent risk factor of Alzheimer's disease (AD). Therefore, identifying periphery biomarkers correlated with mild cognitive impairment (MCI) is of importance for early diagnosis of AD. Here, we performed platelet proteomics in T2DM patients with MCI (T2DM‐MCI) and without MCI (T2DM‐nMCI). Pearson analysis of the omics data with MMSE (mini‐mental state examination), Aβ1‐42/Aβ1‐40 (β‐amyloid), and rGSK‐3β(T/S9) (total to Serine‐9‐phosphorylated glycogen synthase kinase‐3β) revealed that mitophagy/autophagy‐, insulin signaling‐, and glycolysis/gluconeogenesis pathways‐related proteins were most significantly involved. Among them, only the increase of optineurin, an autophagy‐related protein, was simultaneously correlated with the reduced MMSE score, and the increased Aβ1‐42/Aβ1‐40 and rGSK‐3β(T/S9), and the optineurin alone could discriminate T2DM‐MCI from T2DM‐nMCI. Combination of the elevated platelet optineurin and rGSK‐3β(T/S9) enhanced the MCI‐discriminating efficiency with AUC of 0.927, specificity of 86.7%, sensitivity of 85.3%, and accuracy of 0.859, which is promising for predicting cognitive decline in T2DM patients.

## Graphical abstract

Based on proteomic analysis, Yu et al. obtained first‐hand data of platelets in T2DM patients with or without MCI, revealed multiple pathways and proteins correlated to the cognitive decline in T2DM patients. The high efficiency of platelet combination biomarkers was identified in the validation cohort.

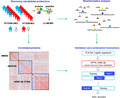

## 1 INTRODUCTION

Type 2 diabetes mellitus (T2DM) and Alzheimer's disease (AD) are age‐related disorders that affect millions of populations worldwide (Chornenkyy et al., 2019; Exalto et al., 2012). Increasing epidemiological data suggest that T2DM is an independent risk factor for AD (Huang et al., 2014; Janson et al., 2004; Strachan et al., 2011). It is also shown that T2DM patients have an increased risk of dementia (73%) compared to non‐T2DM patients, and the cognitive decline seems to begin in the insulin resistance stage of prediabetes (Biessels et al., 2006; Koekkoek et al., 2015). Because of the lifestyle changes, such as diet, overweight and lack of exercise, the incidence of T2DM is rapidly increasing in recent years (Carracher et al., 2018; Kahn et al., 2014). T2DM and AD have many commonalities in pathophysiology, such as amyloidosis, oxidative stress, endothelial dysfunction, and abnormal enzyme activities (de Matos et al., 2018). It is believed that the increasing incidence of AD may be not only related to aging but also to the increasing diabetes (Prince et al., 2013).

Mild cognitive impairment (MCI) is another independent risk factor of AD. Populations with MCI generally developed into AD after decades, which provides a valuable window period for the intervention (Hodson, 2018). Aβ deposition and neurofibrillary tangles formed by the phosphorylated tau proteins are the main pathological features of AD (Jack et al., 2010). However, the accumulation of Aβ has already appeared 10–15 years before the appearance of the clinical phenotypes (Hodson, 2018). The cerebrospinal cord (CSF) level of Aβ1‐42, a marker of amyloidosis, and the level of Aβ‐PET are recognized as effective diagnostic biomarkers for AD (Hansson et al., 2019). However, these methods are invasive or expensive, so that they are hardly popularized in the clinic.

Many evidences suggest that platelets, the fragments shed by megakaryocytes, have many biological similarities with neurons (Chornenkyy et al., 2019; Veitinger et al., 2014). For instance, level of MAO‐B, which is closely related to neuronal activity, is increased significantly in AD platelets (Forlenza et al., 2011). It is also reported that CD62P (P‐selectin) in platelets is activated in AD patients (Sevush et al., 1998), while thrombin receptor activating peptide 6 (TRAP‐6), a molecule related to platelet activation, is decreased in AD (Jaremo et al., 2013). Interestingly, like peripheral synaptic vesicles, platelets share many of the same secretory pathways and transporters as the synaptic terminals of neurons during neurotransmitter uptake and packaging (Kaneez & Saeed, 2009; Walther et al., 2003). The amyloidosis‐related protein BACE1 and tau hyperphosphorylation related protein glycogen synthase kinase‐3β (GSK‐3β), were significantly activated in AD platelets (Colciaghi et al., 2002; Veitinger et al., 2014). We have also reported that the platelet GSK‐3β activity is increased in T2DM with MCI (T2DM‐MCI) patients compared to T2DM without MCI (T2DM‐nMCI) (Z. P. Xu et al., 2016). Therefore, platelets contain abundant information related to the central system and are stable in the peripheral region, which makes it a perfect model for exploring the peripheral biomarkers.

Proteomics is widely used in neuroscience (Bader et al., 2020; Xiong et al., 2019), due to its unique value in deciphering complex pathological mechanisms and screening diagnostic biomarkers. In the present study, we performed an in‐depth and comprehensive proteomic analysis in T2DM‐MCI and T2DM‐nMCI patients. We found that mitophagy/autophagy, insulin signaling, and glycolysis/gluconeogenesis pathways‐related proteins were most significantly deregulated in T2DM‐MCI patients with elevated levels of platelet rGSK‐3β and Aβ1‐42/Aβ1‐40 ratio. The increase of optineurin (OPTN) alone can discriminate T2DM‐MCI from T2DM‐nMCI, and combination of the elevated platelet OPTN with rGSK‐3β has greatly increased the discriminating efficiency.

## 2 RESULTS

### 2.1 Participants information and their platelet protein network alterations during progression of T2DM to MCI

The platelets from two cohorts of T2DM patients were collected for candidate biomarkers screening (10 cases T2DM‐nMCI, 9 cases T2DM‐MCI) and their validation (30 cases T2DM‐nMCI, 34 cases T2DM‐MCI), respectively (Table 1).

**TABLE 1:** Information for T2DM‐MCI and T2DM‐nMCI patients

Characteristic	Proteomics‐Discover set		*p*‐value	Western blot‐Validation set		*p*‐value
	T2DM‐nMCI (*n *= 10)	T2DM‐MCI (*n *= 9)		T2DM‐nMCI (*n *= 30)	T2DM‐MCI (*n *= 34)	
Age, mean (SD), year	71.30 (2.67)	73.3 (5.41)	0.305	63.67 (5.62)	64.47 (6.92)	0.615
Sex (male, female)	4 M, 6F	3 M, 6F	0.764	15 M, 15F	12 M, 22F	0.312
Olfactory score	6.80 (1.03)	7.67 (1.94)	0.233	7.50 (1.43)	8.32 (1.36)	0.022
HbA1c	8.53 (1.99)	7.73 (1.41)	0.376	7.84 (2.05)	8.11 (1.55)	0.591
Diabetes duration, year	11.80 (5.33)	14.00 (8.29)	0.496	7.87 (6.37)	7.24 (6.09)	0.687
Insulin treatment, n (%)	5 (50.0%)	6 (66.7%)	0.653	15 (50.0%)	10 (29.4%)	0.125
Diabetic complications, n (%)	6 (60.0%)	4 (44.4%)	0.498	17 (56.7%)	19 (55.9%)	>0.999
Hypertension, n (%)	5 (50.0%)	8 (88.9%)	0.069	15 (50.0%)	17 (50.0%)	>0.999
Hyperlipidemia, n (%)	4 (40.0%)	5 (55.6%)	0.498	16 (53.3%)	13 (38.2)	0.315
CHD, n (%)	4 (40.0%)	4 (44.4%)	0.845	6 (20.0%)	9 (26.5%)	0.571
APOE ε2 (+), n (%)	2 (20.0%)	0 (0.0%)	0.474	3 (10.0%)	6 (17.6%)	0.483
APOE ε3 (+), n (%)	10 (100%)	9 (100%)	>0.999	30 (100%)	30 (88.2%)	0.116
APOE ε4 (+), n (%)	0 (0.0%)	0 (0.0%)	>0.999	3 (10.0%)	7 (20.6%)	0.313
GSK−3β (Total) (SD)	2.62 (2.30)	2.99 (2.90)	0.760	2.15 (1.83)	2.75 (1.99)	0.218
GSK−3β (S9) (SD)	3.90 (4.11)	2.09 (1.67)	0.237	4.37 (4.51)	2.26 (2.63)	0.024
rGSK−3β (Total/S9) (SD)	0.76 (0.21)	3.52 (5.38)	0.122	0.66 (0.38)	2.09 (2.70)	0.006
Aβ1‐40 (SD)	268.50 (117.70)	95.56 (102.80)	0.004	194.50 (95.17)	177.80 (95.92)	0.487
Aβ1‐42 (SD)	63.51 (24.66)	74.23 (29.44)	0.400	61.54 (21.68)	61.90 (20.40)	0.945
Aβ1‐42/1‐40 (SD)	0.32 (0.28)	1.40 (1.06)	0.006	0.40 (0.27)	0.60 (0.71)	0.165
MMSE	28.70 (0.67)	20.11 (3.41)	<0.001	28.67 (0.71)	22.97 (2.53)	<0.001

Abbreviations: APOE, Apo lipoprotein E; CHD, Coronary heart disease; GSK‐3β, glycogen synthase kinase‐3β; HbA1c, hemoglobin A1c; MCI, mild cognitive impairment; MMSE, the Minimum Mental State Examination; rGSK3β, GSK‐3β‐total/GSK‐3β‐S9; T2DM, type 2 diabetes mellitus; T2DM‐MCI, T2DM with MCI group; T2DM‐nMCI, T2DM without MCI group.

**FIGURE 1:**
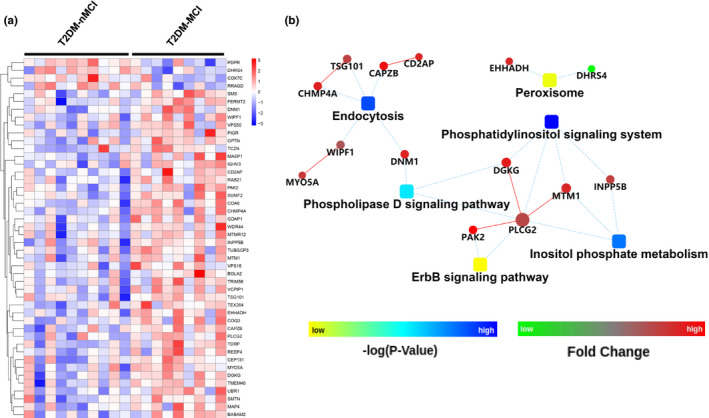
Differentially expressed proteins (DEPs) and protein‐protein interaction (PPI) networks in T2DM‐MCI vs T2DM‐nMCI. (a) 46 DEPs were identified in the platelet of T2DM‐MCI patients compared with the T2DM‐nMCI (*p* < 0.05, increased proteins: red; decreased proteins: blue). (b) The PPI networks indicate the interactions of DEPs with each other in T2DM‐MCI vs T2DM‐nMCI, and endocytosis, peroxidase, ErbB, and phosphatidylinositol signaling system are closely associated

By using TMT‐LC‐MS/MS proteomics, a total of 2994 platelet proteins were captured, of which 46 differentially expressed proteins (DEPs) were identified in T2DM‐MCI *vs*. T2DM‐nMCI (*p* < 0.05) (Figure 1a, Excel S1). To further understand the biological function of DEPs and the signaling events, PPI network analysis was performed based on KEGG database. As shown in Figure 1b, the complex network regulation of the DEPs was mainly involved in endocytosis, peroxidase, ErbB, phosphatidylinositol signaling pathways.

**FIGURE 2:**
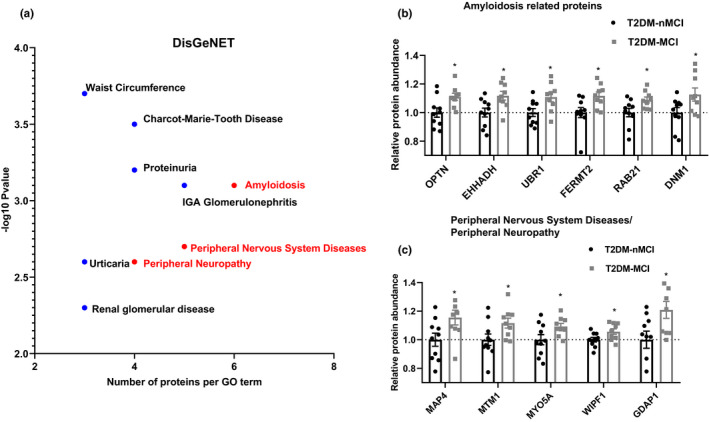
Integrating information on human disease‐associated DPEs. (a) GO term of top 8 gene disease network (DisGeNET) with ‐log10 (*p*‐*value*). (b, c) Relative expression abundance of amyloidosis and peripheral neuropathy, peripheral nervous system diseases. *p < 0.01 vs. the T2DM‐nMCI subjects

Based on metascape online analysis software, the top 8 gene disease network (DisGeNET) with ‐log10 (*p*‐*value*) was obtained, including amyloidosis, peripheral neuropathy and peripheral nervous system diseases (Figure 2a). Specifically, the proteins involved in amyloidosis, including optineurin (OPTN), ras‐related protein Rab‐21 (Rab21), dynamin 1 (DNM1), peroxisomal bifunctional enzyme (EHHADH), fermitin family homolog 2 (FERMT2), E3 ubiquitin‐protein ligase UBR1 were increased in T2DM‐MCI compared with T2DM‐nMCI (Figure 2b). The peripheral nervous system diseases related proteins, such as microtubule‐associated protein 4 (MAP4), myotubularin (MTM1), unconventional myosin‐Va (MYO5A), WAS/WASL‐interacting protein family member 1 (WIPF1), and ganglioside‐induced differentiation‐associated protein 1 (GDAP1) were also increased in T2DM‐MCI compared with T2DM‐nMCI (Figure 2c).

These data together reveal the complex platelet protein network alterations during progression of T2DM to MCI, providing a valuable resource for their systematic discovery and validation.

### 2.2 Potential platelet biomarkers associated with MCI in T2DM patients

**FIGURE 3:**
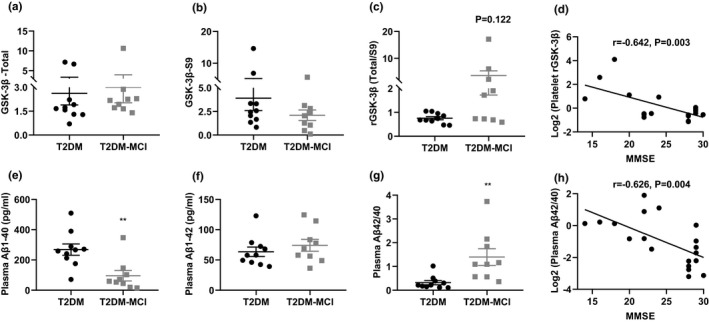
Levels of platelet rGSK3β(T/S9) and plasma Aβ1‐42/Aβ1‐40 in T2DM‐nMCI and T2DM‐MCI patients. (a‐c) Comparison of GSK‐3β‐Total, GSK‐3β‐S9 and rGSK‐3β (Total/S9) in the platelet of T2DM‐nMCI and T2DM‐MCI patients. (e‐g) Comparison of Aβ1‐40, Aβ1‐42 and Aβ1‐42/Aβ1‐40 in the plasma of T2DM‐nMCI and T2DM‐MCI patients. **p < 0.01 vs. the T2DM‐nMCI subjects. (d, h) Correlation between rGSK‐3βor Aβ1‐42/Aβ1‐40 and MMSE (rGSK‐3β and MMSE: *r* = −0.642, *p* = 0.003; Aβ1‐42/Aβ1‐40 and MMSE: *r* = −0.626, *p* = 0.004)

During patient selection for this proteomic analysis, we payed special attention to ApoE genetype, olfactory function, and rGSK‐3β(T/S9), because changes of these factors are correlated to the cognitive decline in T2DM patients (Michaelson, 2014; Rahayel et al., 2012; Zhang et al., 2018). Although a strong trend of increase was shown for platelet rGSK‐3β(T/S9), the difference did not reach statistical significance between T2DM‐MCI and T2DM‐nMCI groups (GSK‐3β‐Total: *p* = 0.760; GSK‐3β: *p* = 0.237; rGSK‐3β: *p* = 0.122) (Figure 3a‐c). No significant difference was shown for ApoE genetype or olfactory function between the two groups (Table 1), which may be due to the relatively small sample size. We also detected plasma levels of Aβ1‐40 and Aβ1‐42, because the β‐amyloidosis is the common pathology in both AD and diabetes patients (de Matos et al., 2018). We found that Aβ1‐40 was decreased in T2DM‐MCI compared with T2DM‐nMCI patients while no significant difference was detected for Aβ1‐42 (*p* = 0.400), consequently, the ratio of Aβ1‐42/Aβ1‐40 was increased in T2DM‐MCI vs T2DM‐nMCI (Figure 3e‐g). Furthermore, a negative correlation between MMSE score and ratio of Aβ1‐42/Aβ1‐40 was shown (*r* = −0.626, *p* = 0.004; Figure 3h)). Though the increase of rGSK‐3β was not statistically significant (Figure 3a‐c), we also detected a negative correlation between MMSE score and rGSK‐3β (Figure 3d). Therefore, we also employed Aβ1‐42/Aβ1‐40 and rGSK‐3β as the parameters for platelet new biomarker screening in addition to MMSE score.

**FIGURE 4:**
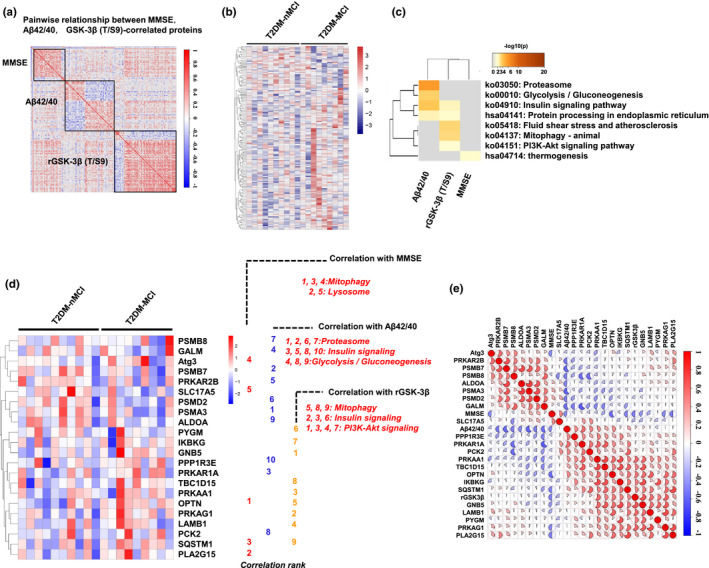
Proteins correlated with MMSE, Aβ1‐42/Aβ1‐40, and rGSK‐3β(T/S9) in platelet proteomics. (a) Correlation analysis of MMSE, Aβ1‐42/Aβ1‐40, and rGSK‐3β to the entire omics data analyzed by Pearson (*p* < 0.05) (positive correlation, red; negative correlation, blue). (b) Heatmap of the relative abundance of all MMSE, Aβ1‐42/Aβ1‐40, and rGSK‐3β‐correlated proteins in each sample (increased proteins: red; decreased proteins: blue). (c) Enriched KEGG pathway of all correlated proteins (the significance of the enriched pathway was defined as overlap proteins ≥3, *p* < 0.01). (d) KEGG pathway enriched by MMSE, Aβ1‐42/Aβ1‐40, and rGSK‐3β‐correlated proteins. All proteins involved in the pathway were ranked according to their correlation coefficient. MMSE‐correlated proteins were mainly involved in mitophagy (1,3,4) and lysosome (2, 5). Aβ1‐42/Aβ1‐40‐correlated proteins were mainly involved in proteasome (1, 2, 6, 7), insulin signaling (3, 5, 8, 10) and glycolysis or gluconeogenesis (4, 8, 9). The rGSK‐3β‐correlated proteins were mainly involved in mitophagy (5, 8, 9), insulin signaling (2, 3, 6) and PI3K‐Akt signaling (1, 3, 4, 7). (e) The correlation matrix of all proteins involved in panel d

By Pearson analysis, a total of 150 correlated proteins were identified in T2DM‐MCI vs T2DM‐nMCI groups, among which MMSE, Aβ1‐42/Aβ1‐40 and rGSK‐3β‐correlated proteins were respectively accounted for 40, 53, and 65 with *r *values of 0.456–0.726 (Figure 4a and Excel S2) and relative abundance values shown in Figure 4b and Excel S3. The KEGG pathway analyses showed that the MMSE‐correlated proteins (*n* = 40) were mainly related to mitophagy (OPTN, SQSTM1 and Atg3), and lysosome, such as solute carrier family 17 member 5 (SLC17A5) and phospholipase A2 group XV (PLA2G15) (Figure 4c‐d). The Aβ1‐42/Aβ1‐40‐correlated proteins were mainly involved in glycolysis, gluconeogenesis, and insulin signaling pathway (*n* = 53), such as proteasome subunit alpha type‐3 (PSMA3), cAMP‐dependent protein kinase type I‐alpha regulatory subunit (PRKAR1A), phosphoenolpyruvate carboxykinase [GTP] (PCK2), fructose‐bisphosphate aldolase A (ALDOA) (Figure 4c‐d). The rGSK‐3β‐correlated proteins (*n* = 65) were mainly related to mitophagy, insulin, and PI3K‐Akt signaling pathways (Figure 4c‐d).

To describe the signal transduction pathway more intuitively, cytoscape (3.7.0) software and its wiki pathway, KEGG plug‐in were used to specifically display the signal regulation pathway. As shown in Figure S1a, TBC1D15 is involved in autophagy encapsulation of mitochondria, while OPTN and SQSTM1 (P62) can bind to ubiquitination substrates and promote protein degradation. Additionally, PKA regulation subunits, PRKAR1A and PRKAR2B, were correlated with Aβ1‐42/Aβ1‐40, and AMPK subunit PRKAA1, PRKAG1 correlated with rGSK3β, which are involved lipid metabolism in the insulin signaling pathway (Figure S1b).

All proteins involved in the pathways were ranked according to their correlation coefficient (Figure 4d), and the relative abundance of each protein in each sample is represented by a heat map (Figure 4d and Excel S4). By integrating correlated proteins involved in the above signaling pathways, we obtained a mixed correlation matrix combining pathological/clinical features and proteins (Figure 4e and Excel S5). Interestingly, we found some closely correlated proteins in the data list, such as mitophagy proteins OPTN, SQSTM1, and TBC1D15 (*r* = 0.50–0.61) (Figure 4e), which were also widely correlated with the proteins enriched in PI3K‐Akt signaling pathway (*r* = 0.46–0.71). In addition, OPTN and SQSTM1 were also strongly correlated with PRKAR1A (*r* = 0.59) and PRKAA1 (*r* = 0.59) in AMPK subunit (Figure 4e and Figure S2).

These data together suggest that proteins associated with deregulated mitophagy/autophagy, insulin signaling, and glycolysis/gluconeogenesis pathways could be potential platelet biomarkers for cognitive decline in T2DM patients.

### 2.3 OPTN elevation in identifying MCI from T2DM patients

**FIGURE 5:**
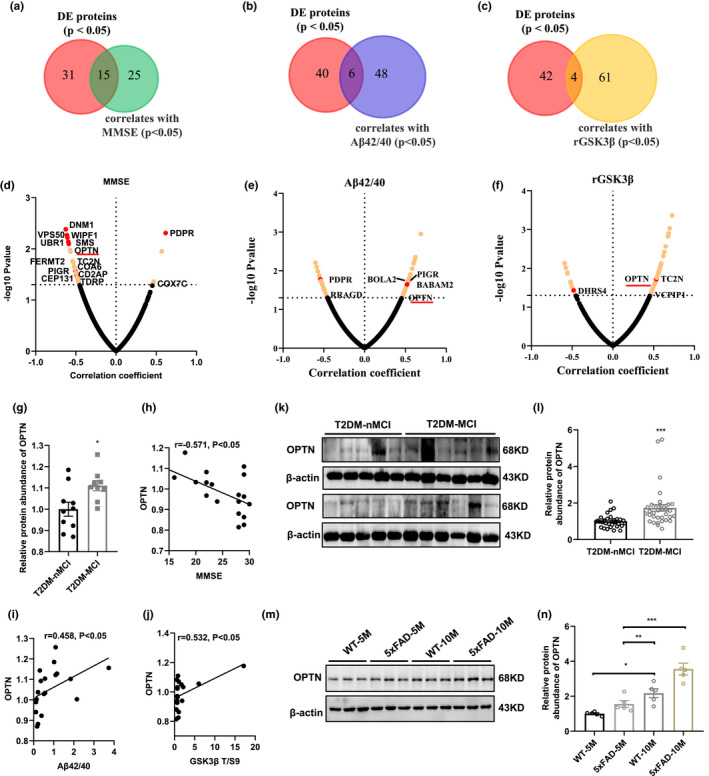
Elevated platelet OPTN can discriminate T2DM‐MCI from T2DM‐nMCI. (a‐c) Overlap of proteins significantly different in T2DM‐MCI *vs*. T2DM‐nMCI with proteins correlated to MMSE, Aβ1‐42/Aβ1‐40, and rGSK‐3β (*p* < 0.05). (d‐f) Correlation of proteins with MMSE (d), Aβ1‐42/Aβ1‐40 (e), and rGSK3β (f). Proteins with corresponding *p values* <0.05 are labeled in yellow. Overlap of proteins have been marked in red dot. OPTN is the only protein correlated with MMSE, Aβ1‐42/Aβ1‐40, and rGSK‐3β. (g) Dot plots represent the relative expression level of OPTN in different samples (*p* < 0.05). (h‐j) Correlation of OPTN with MMSE (h; *r* = −0.571, *p* = 0.011), Aβ1‐42/Aβ1‐40 (i; *r* = 0.458, *p* = 0.049), and rGSK‐3β (j; *r* = 0.532, *p* = 0.019). (k, l) Relative levels of platelet OPTN in T2DM‐MCI compared to T2DM‐nMCI. Data were shown as mean ±SEM. ***p < 0.001; T2DM‐nMCI: *n* = 30; T2DM‐MCI: *n* = 34. (m, n) Relative levels of hippocampal OPTN in 5‐ and 10‐month old 5xFAD mice compared to age‐matched WT mice. Data were shown as mean ±SEM. *p < 0.05, **p < 0.01 and ***p < 0.001, *n* = 5

Next, we performed venn logic analysis to determine the correlation of the DEPs with MMSE (*n* = 15), Aβ1‐42/Aβ1‐40 (*n* = 6), and rGSK‐3β (*n* = 4), respectively (Figure 5a‐f). The MMSE‐correlated DEPs were mainly involved in amyloidosis (DNM1, UBR1, OPTN, FERMT2), CNS disorder (WIPF1) and energy metabolism (COA6, COX7C, PDPR) processes (Figure 5a and d). The Aβ1‐42/Aβ1‐40‐correlated DEPs included OPTN, pyruvate dehydrogenase phosphatase regulatory subunit (PDPR), Ras‐related GTP‐binding protein D (RRAGD), BolA‐like protein 2 (BOLA2), polymeric immunoglobulin receptor (PIGR), BRISC and BRCA1‐A complex member 2 (BABAM2) (Figure 5b and e). The rGSK‐3β‐correlated DEPs included OPTN, dehydrogenase/reductase SDR family member 4 (DHRS4), receptor protein serine/threonine kinase (TC2N), deubiquitinating protein VCIP135 (VCPIP1) (Figure 5c and f).

We found excitingly that among the above DEPs, only the elevated OPTN (Figure 5g) was significantly correlated with MMSE (*r* = −0.571, *p* = 0.011; Figure 5h), Aβ1‐42/Aβ1‐40 (*r* = 0.458, *p* = 0.049; Figure 5i), and rGSK‐3β (*r* = 0.532, *p* = 0.019; Figure 5j) in T2DM‐MCI patients, suggesting a strong power of OPTN as a biomarker for identifying MCI in T2DM patients. Studies have shown that the OPTN‐mediated autophagy pathway is closely related to the degradation of Aβ and Tau proteins (Du et al., 2017; Y. Xu et al., 2019).

To further verify whether the above predicted OPTN elevation can be used as a biomarker for the clinic to identify MCI in T2DM patients, we analyzed the OPTN by Western blotting. Consistent with the proteomic results, the level of platelet OPTN was significantly increased in T2DM‐MCI patients (*n* = 34) compared with the T2DM‐nMCI patients (*n* = 30) (Figure 5k‐l). An age‐dependent elevation of OPTN was also detected in 5xFAD and the control mice (Figure 5m‐n).

### 2.4 Combined platelet OPTN and rGSK3β elevation in discriminating T2DM‐MCI from T2DM‐NMCI patients

**FIGURE 6:**
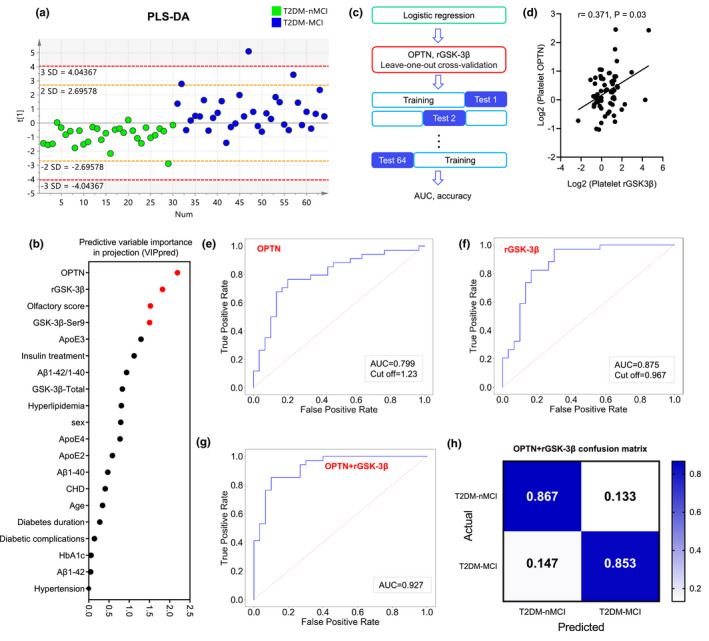
Combined platelet OPTN and rGSK3β discriminates T2DM‐MCI from T2DM‐nMCI with high efficiency. (a) Discrimination power of the patient information and OPTN analyzed by PLS‐DA analysis. (b) OPTN, rGSK‐3β, olfactory score, and GSK‐3β‐Ser9 (VIP>1.5) were selected by predictive variable importance in projection (VIPpred) analysis (Red plot: VIP >1.5; Black plot: VIP <1.5). (c) The workflow of machine‐learning strategy: logistic regression and leave‐one‐out (LOO) cross‐validation were used to test the diagnostic efficiency of potential combination biomarkers. (d) Correlation of OPTN with rGSK‐3β (*r* = 0.371, *p* = 0.03). (e‐g) AUC values for discriminating efficiency of T2DM‐MCI from T2DM‐nMCI with OPTN (e), rGSK‐3β (f) and in combination of OPTN and rGSK‐3β (g) analyzed by LOO algorithm. (h) The confusion matrix of the combined biomarkers

By using PLS‐DA analysis, we further identified the contribution of different variables in discriminating MCI in T2DM patients (Figure 6a). The results showed that four factors, that is OPTN, rGSK‐3β, olfactory score, and GSK‐3β‐Ser9, have the greatest contribution in distinguishing T2DM‐MCI from the T2DM‐nMCI patients (Figure 6b).

By logistic regression algorithm analysis, we further calculated the significance level of the coefficient (Sig.) and 95% confidence intervals (95% CI) for OPTN, rGSK‐3β, olfactory score, and GSK‐3β‐Ser9 (Figure 6c). We found that OPTN and rGSK‐3β (Sig. <0.05) were strongly associated with MCI in T2DM patients (Table S1). Leave‐one‐out (LOO) cross‐validation was used to test the diagnostic efficiency of OPTN and rGSK‐3β in discriminating MCI from T2DM patients (Figure 6c). A positive correlation between the elevated OPTN and rGSK‐3β was detected (*r* = 0.371, *p* = 0.03; Figure 6d), in which the efficiency of the elevated rGSK‐3β in identifying MCI in T2DM patients had been reported in our previous study (Z. P. Xu et al., 2016). Machine‐learning results showed that both the elevated platelet OPTN (AUC = 0.799, accuracy = 76.6%, cut off = 1.23, sensitivity = 70.6% and specificity = 83.3%) and rGSK‐3β (AUC = 0.875, accuracy = 78.1%, cut off = 0.967, sensitivity = 73.5% and specificity = 83.3%) could efficiently discriminate T2DM‐MCI from T2DM‐nMCI patients in validation set (Figure 6e‐f and Figure S3).

By combining OPTN and rGSK‐3β, we generated a ROC curve with an AUC of 0.927 and accuracy of 85.9%, sensitivity of 85.3%, and specificity of 86.7% (Figure 6g) in the validation set (Figure 6h). These data indicate that combining the elevated platelet OPTN and rGSK‐3β can most efficiently distinguish T2DM‐MCI from T2DM‐nMCI patients.

## 3 DISCUSSION

Type 2 diabetes mellitus (T2DM) is an independent risk factor for AD (Huang et al., 2014; Janson et al., 2004; Strachan et al., 2011), therefore, predicting who, in T2DM populations, will suffer from dementia is important for early diagnosis and intervention of AD. By employing a highly sensitive TMT‐LC‐MS/MS, bioinformatics and machine learning, we carried out a comprehensive proteomic analysis in T2DM‐MCI (*n* = 9) and T2DM‐nMCI (*n* = 10) patients. A total of 4165 proteins were identified, of which 2994 were captured in each group. Further analysis demonstrated that the significantly altered platelet proteins were mainly involved in endocytosis, phosphatidylinositol signaling system, amyloidosis and peripheral nervous system, which could be the target pathways for the cognitive decline in T2DM patients. These data provide a valuable resource for exploring potential periphery platelet biomarkers and the molecular mechanisms underlying the cognitive impairments in T2DM patients.

We have recently demonstrated that platelet rGSK‐3β elevation, olfactory dysfunction and APOE ε4 genetype were positively correlated to the cognitive decline in T2DM patients (Z. P. Xu et al., 2016). Here, we did not find statistical difference in these factors in proteomics screening cohort, which may be due to the relatively small sample size used for the proteomics. In the validation cohort, we found a significantly increased rGSK‐3β in T2DM‐MCI group with a negative correlation to MMSE score. As β‐amyloidosis is comorbidity for both T2DM and AD, we measured plasma Aβ level. A significantly elevated Aβ1‐42/Aβ1‐40 was detected in the proteomics cohort with a negative correlation to the reduced MMSE score. Therefore, we brought MMSE, Aβ1‐42/Aβ1‐40 and rGSK‐3β into the following new biomarker studies. By which, we discovered that the significantly changed proteins were mainly enriched in the deregulated mitophagy/autophagy pathway (OPTN, SQSTM1, TBC1D15), insulin signaling pathway (PRKAR1A, PRKAR2B, PRKAA1, PRKAG1), and glycolysis/gluconeogenesis pathway (GALM, PCK2, ALDOA). Among them, OPTN is the only differentially expressed protein correlated with all three factors (i.e., decreased MMSE score, increased Aβ1‐42/Aβ1‐40 and rGSK‐3β), and elevation of OPTN showed a strong power in discriminating T2DM‐MCI from T2DM‐nMCI patients.

OPTN is an autophagy receptor, which links ubiquitinated substrates to autophagy membrane, and thereby mediates PINK1‐driven clearance of the damaged mitochondria (Richter et al., 2016). OPTN also mediates clearance of Aβ (Du et al., 2017) and soluble tau through autophagy pathway, while SQSTM1, another autophagy receptor, targets clearance of insoluble tau proteins (Y. Xu et al., 2019). We found in the current study that OPTN was significantly increased in platelets of T2DM‐MCI patients and the hippocampus of aged 5xFAD, which was also observed in the brain of AD patients (Cho et al., 2014). PRKAA1 can mediate the binding of ubiquitin substrate linked with OPTN to MAP1LC3, by which it promotes autophagy degradation (Cho et al., 2014). It is well recognized that dysfunction of autophagy pathway leading to Aβ and tau accumulation plays a pivotal role in the chronic progression of AD pathologies (Nixon & Cataldo, 2006) (Zare‐Shahabadi et al., 2015) (Fang et al., 2019). Tau accumulation can in turn aggravate autophagy deficit which forms a vicious cycle, and the autophagosome‐lysosome fusion deficit caused by tau accumulation induces autophagy flow inhibition (Feng et al., 2020). Based on these observations, we speculate that inhibition of autophagy flow may be involved in OPTN‐related cognitive decline, though the detailed mechanisms need further investigation.

In addition to OPTN, changes of FEMT2, RAB21, DNM1 were also observed in T2DM‐MCI group. FERMT2 is a high‐risk gene for AD (Karch & Goate, 2015), and epidemiological data show that it is stage‐dependently associated with brain amyloidosis, and most significant in MCI (Apostolova et al., 2018). RAB21 is mainly involved in the process of endocytosis and autophagy, and it can promote γ‐secretase internalization and translocation to the endosome/lysosome, and thus exacerbate Aβ production in AD (Sun et al., 2018). Increased levels of mitochondrial fission‐associated protein DNM1 promotes mitochondrial fragmentation, mitochondrial dynamics disorder, and thus exacerbating Aβ clearance disorder (Manczak et al., 2011). Therefore, further validation of these proteins in larger populations will confirm their role to be a periphery biomarker for predicting cognitive decline in T2DM patients.

It is well known that T2DM patients always show peripheral nervous damages, such as microangiopathy (Zochodne, 2007). We also observed that proteins enriched in GO term of peripheral nervous system disease, including MAP4, MTM1, MYO5A and GDAP1, were significantly increased in T2DM‐MCI patients. As a family member of microtubule‐associated proteins, MAP4 plays a role in stabilizing microtubules, but it is not expressed in neurons (Nguyen et al., 1997). MTM1 is primarily involved with congenital myopathies through phosphatidylinositol signaling (Blondeau et al., 2000). MYO5A is highly expressed in the brain mainly at synapses, where it promotes the transport of AMPA glutamate receptors to the synapse and participating in the development of the synapse (Ultanir et al., 2014). GDAP1 is highly expressed in neurons and localized in the outer membrane of mitochondria, which mainly affects mitochondrial dynamics, mitochondrial distribution along axons and oxidative stress process (Gonzalez‐Sanchez et al., 2019). How these proteins were transported between the brain and the periphery platelet deserves further investigation. According to the previous report (Reinhold & Rittner, 2017), we speculate that destruction of nerve barrier or brain blood barrier during T2DM progression may be involved.

Consistent with our previous findings that rGSK‐3β has the highest efficiency in identifying MCI from T2DM patients, compared with other characteristic factors, such as aging, ApoE genetype, and olfaction (Z. P. Xu et al., 2016), we confirmed the role of rGSK‐3β in the current study. We further identified that the rGSK‐3β‐correlated proteins were enriched in the AD‐related lipid metabolism (PRKAA1, PRKAG1) and mitophagy/autophagy pathways (OPTN, SQSTM1, TBC1D15). The elevated OPTN was positively correlated with rGSK‐3β in validation set. Furthermore, combination of the elevated platelet OPTN and rGSK‐3β remarkably enhanced the MCI‐discriminating efficiency from T2DM patients with AUC of 0.927 and accuracy of 0.859. In future studies, such an incredible combination will be verified on larger cohorts.

In summary, recent brain/CSF proteomic data show that autophagy pathways, glucose metabolism, and amyloidosis‐related proteins are significantly dysregulated in AD patients (Bai et al., 2020; Johnson et al., 2020; Wang et al., 2020). By an in‐depth and comprehensive platelet proteomic analysis in T2DM‐MCI vs T2DM‐nMCI patients, we demonstrated that the differentially expressed proteins were mainly enriched in amyloidosis, mitophagy/autophagy and insulin signaling pathways. Machine learning further identified OPTN and rGSK‐3β as the most distinctive MCI‐related platelet biomarkers. In addition, this study has also provided the first‐hand data on the understanding of platelets in T2DM patients with or without MCI. Longitudinal follow‐up studies on large cohort will further validate the efficiency of these periphery biomarkers in predicting who in T2DM population is more vulnerable to AD.

## 4 EXPERIMENTAL PROCEDURES

### 4.1 Participants information

All the type 2 diabetes mellitus (T2DM) patients from the Central Hospital of Wuhan were divided into two groups: the T2DM without mild cognitive impairment (T2DM‐nMCI) group and the T2DM with mild cognitive impairment group (T2DM‐MCI), which met the National Institute on Aging and the Alzheimer's Association Guidelines (Albert et al., 2011), and received mini‐mental State Examination (MMSE) test scores (Folstein et al., 1975) (Table 1). In this study, platelets from two cohorts were used for candidate biomarker screening (10 T2DM‐nMCI, 9 T2DM‐MCI) and validation (30 T2DM‐nMCI, 34 T2DM‐MCI), respectively. All samples excluded traumatic brain injury, brain tumors, drug abuse, alcohol addiction, and psychiatric disorders. Diabetes, hypertension, hyperlipidemia and coronary heart disease (CHD), olfactory score, Apo lipoprotein E (APOE) were considered systematically (Table 1).

The study was approved by the Tongji Medical School Ethics Committee, complies with the Helsinki Declaration II, and includes written informed consent from all participants. The project “Early Detection of Cognitive Dysfunction in Diabetes” was registered in the Chinese Clinical Trial Registry (https://clinicaltrials.gov; NCT01830998).

5×FAD [B6. Cg‐Tg(APPSwFlLon,PSEN1*M146L*L286V)6799V] and the age/sex‐matched wild‐type control mice were purchased from the Jackson Laboratory (Maine, USA). Animals were housed in a 12–12‐h light‐dark cycle environment with unlimited access to drinking water and food. The animal study was also reviewed and approved by Ethics Committee of the Shenzhen Center for Disease Control and Prevention.

### 4.2 Sample preparation

The fresh blood stored in the anticoagulant tube was centrifuged at 200 g for 20 min to remove rich red and white blood cells from the plasma, and 2/3 of the platelets rich supernatant was brought into the new tube and centrifuged at 120 g for 6 min to remove remaining white blood cells, and centrifuged at 1500 g for 10 minutes to obtain relatively pure platelet precipitate. Further, the platelet precipitate was washed with tyrode's solution (143.0 mM NaCl, 5.4 mM KCl, 0.25 mM NaH2PO4, 1.8 mM CaCl2, 0.5 mM MgCl2, 5.0 mM HEPES, pH 7.4; Solarbio, T1420, Beijing, China) and centrifuged at 120g for 4 min to obtain purified platelet samples and stored at −80℃.

The platelet samples were completely lysed by ultrasound (120s, 4 s on and 6 s off) after adding the lysis buffer (8 M urea, pH 8.0, 1 cocktail, 1 mM PMSF), and then lysed on ice for 30 min, and centrifuged at 12000 g for 10 minutes to obtain the pure protein solution.

### 4.3 TANDEM mass TAG (TMT) labeling

After mass spectrometry trypsin (Promega, V5072) cleavage, each TMT label (ThermoFisher 90406) was attached to each sample (*n *= 19). The samples with different TMT labels were mixed together and divided into 15 components by high performance liquid chromatography (HPLC) step by step for subsequent experiments.

### 4.4 Data collection of TMT‐labeled peptides using LC‐MS/MS

The dried components were dissolved in 0.1% formic acid (FA), and captured with a silica gel capillary column filled with C18 resin (Varian, Lexington, MA, USA) for subsequent Q Exactive (Thermo Scientific, NJ, USA) mass spectrometer analysis. Full scan in Orbitrap mass analyzer in data‐dependent acquisition (DDA) mode, the specific parameters are set as follows: 400–1, 800 m/z, 70000 resolution; MS/MS scans (100−1800 m/z). Using Proteome Discoverer 2.1 software (Thermo Scientific) to retrieve MS/MS data according to Uniport‐human database (2020–05). The searching parameters were modified on the previous research settings (B. Xu et al., 2019).

### 4.5 Bioinformatics analysis

Normalized data were uploaded to Perseus platform, and proteins with *p* < 0.05 were evaluated by t‐test and considered differentially expressed (Bereczki et al., 2018). R studio (v.0.99.489) and heatmap gplots package have been widely used for cluster analysis and heatmap drawing. Prism 8.0 is used for volcano map and heatmap analysis. Metascape (http://metascape.org), WEB‐based GEne SeT AnaLysis Toolkit (http://www.webgestalt.org) and DAVID version 6.7 (https://david‐d.ncifcrf.gov/) were used to pathways and functional analysis. Cytoscape 3.6.1 and STRING (v10; https://string‐db.org/) plug‐in were used for visual analysis of protein‐protein interaction (PPI) network. KEGG Mapper–Search & Color Pathway (https://www.kegg.jp/kegg/tool/map_pathway2.html) was used for pathway enrichment analysis.

### 4.6 ELISA and western blotting

Plasma Aβ40 and Aβ42 were detected by a commercial ELISA kit (E‐EL‐H0542c 90 and E‐EL‐H0543c, Elabscience, China). The platelet OPTN level was detected by Western blotting (ab213556, Abcam) routinely used in our laboratory.

### 4.7 Machine learning

SIMCA (version 14.0) software was used for partial least squares discrimination analysis (PLS‐DA). The protein of predictive variable importance in projection larger than 1.5 (VIPpred >1.5) was considered to be meaningful for sample discrimination. Logistic regression (LR), a widely used machine‐learning algorithm, was used to calculate the 95% confidence interval (95% CI) of the biomarker for diagnosis of MCI. The samples were trained and evaluated in a leave‐one‐out (LOO) cross‐validation manner using scikit‐learn python package, which was used for model training and parameter optimization in the life sciences was used for model training and parameter optimization (Bader et al., 2020; Shu et al., 2020). More specifically, 63 samples were randomly selected from 64 samples each time for modeling, and the remaining one was used for validation. Thus, 64 cycles are carried out to achieve the purpose of full data demonstration and cross‐validation. Confusion matrix was used to assess the specificity and sensitivity of biomarkers in the diagnosis of MCI.

### 4.8 Statistical analysis

The data were expressed as mean ±s.e.m. with SPSS 24.0 software (Statistical Program for Social Sciences Inc., Chicago, IL, USA). The student's t‐test was used to evaluate the level of significance between the two groups, and *p values* <0.05 was considered to be significant.

For the workflow of the analytic procedure, please also see Figure S4.

## CONFLICT OF INTEREST

The authors declare that they have no conflict of interest to disclose.

## AUTHOR CONTRIBUTIONS

Experimental design: HY, XY, J‐ZW; recruitment of subjects and sample collection: HY, YL, BH, TH, YG, YZ, DK; experimental methods: HY, YL, TH, CC, XY, J‐ZW, JL, ML; data analysis: HY, YL, JH, CC, BJ, TH; manuscript‐writing: HY, XY, J‐ZW.

## Supporting information

Fig S1‐S3

Fig S4

Excel S1

Excel S2

Excel S3

Excel S4

Excel S5

Table S1

## Data Availability

All data used to support the findings of this study are included within the article. Raw data used to generate the figures are available from Proteome Xchange Consortium (http://www.proteomexchange.org) via the PRIDE partner repository with the dataset identifiers PXD023316.
