# Supplementary material for: Platelet biomarkers identifying mild cognitive impairment in type 2 diabetes patients
Source: Aging Cell. 2021 Sep 16;20(10):e13469. doi: 10.1111/acel.13469 (PMC8520722; doi:10.1111/acel.13469)
Supplement: Supplementary file 1 — Fig S1‐S3 [file ACEL-20-e13469-s005.pdf]

## A. Mitophagy pathway

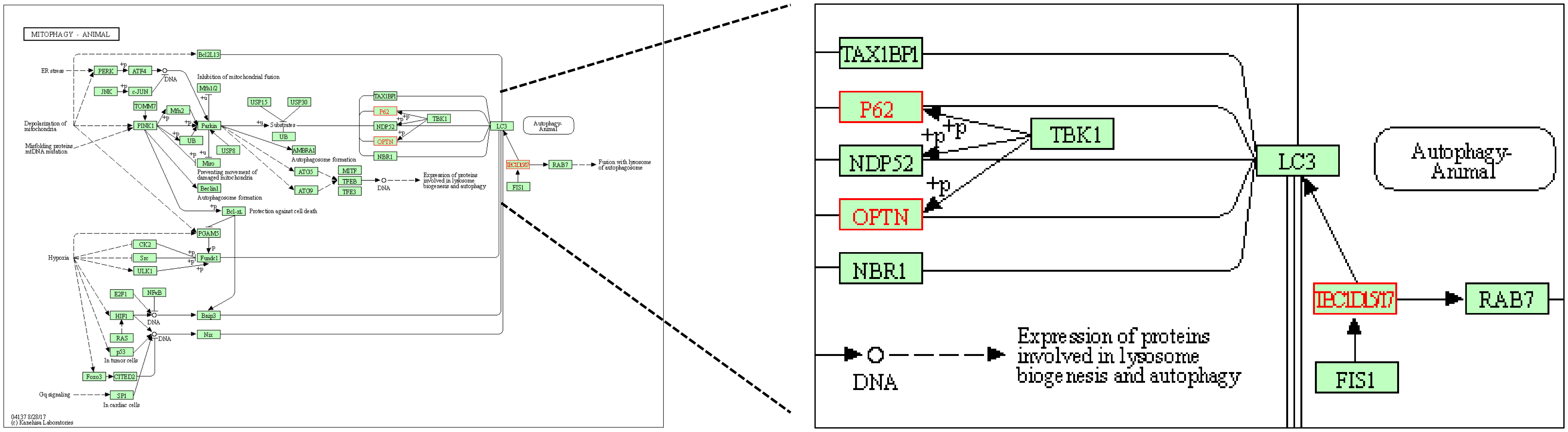


## B. Lipid Metabolism Pathway

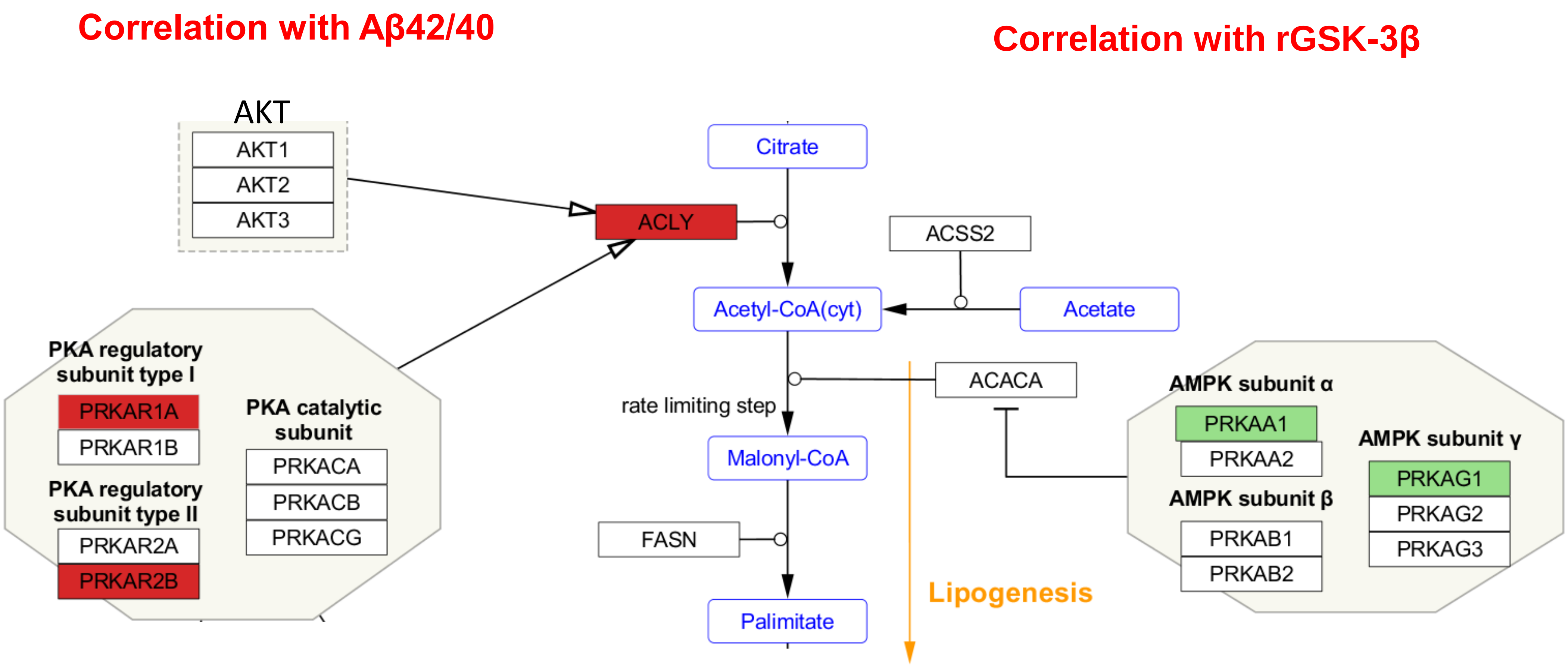


**Figure S1** Yu haitao et al., 2021

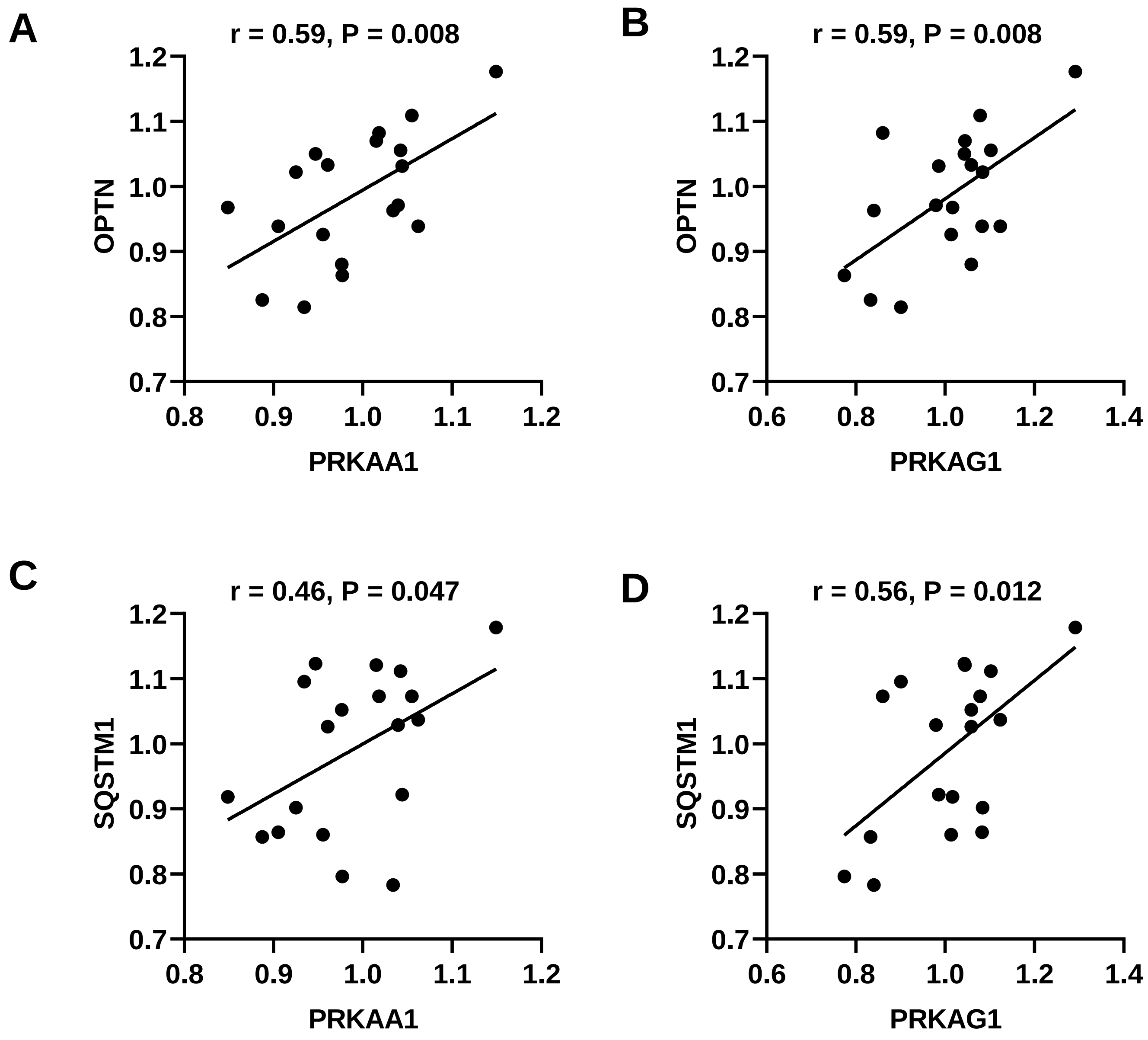


**Figure S2**

Yu haitao et al., 2021

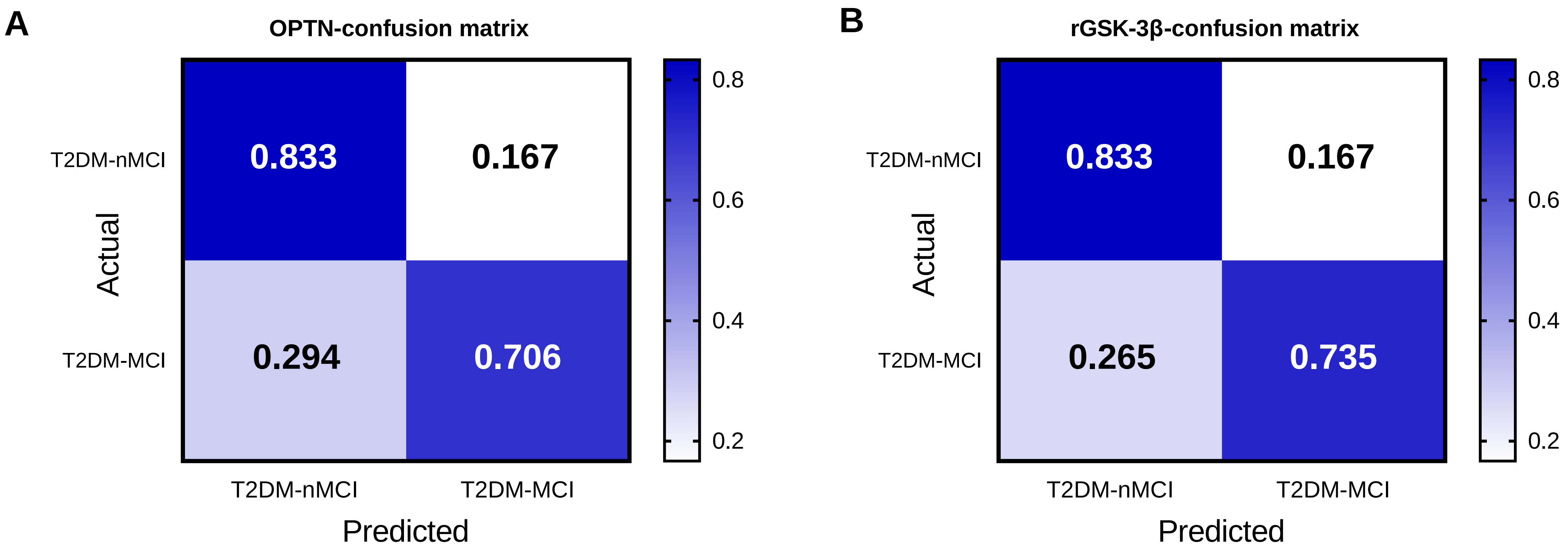


**Figure S3**

**Yu haitao et al., 2021**
