# Supplementary figures and images for: Platelet biomarkers identifying mild cognitive impairment in type 2 diabetes patients
Source: Aging Cell. 2021 Sep 16;20(10):e13469. doi: 10.1111/acel.13469 (PMC8520722; doi:10.1111/acel.13469)

# Graphical Abstract

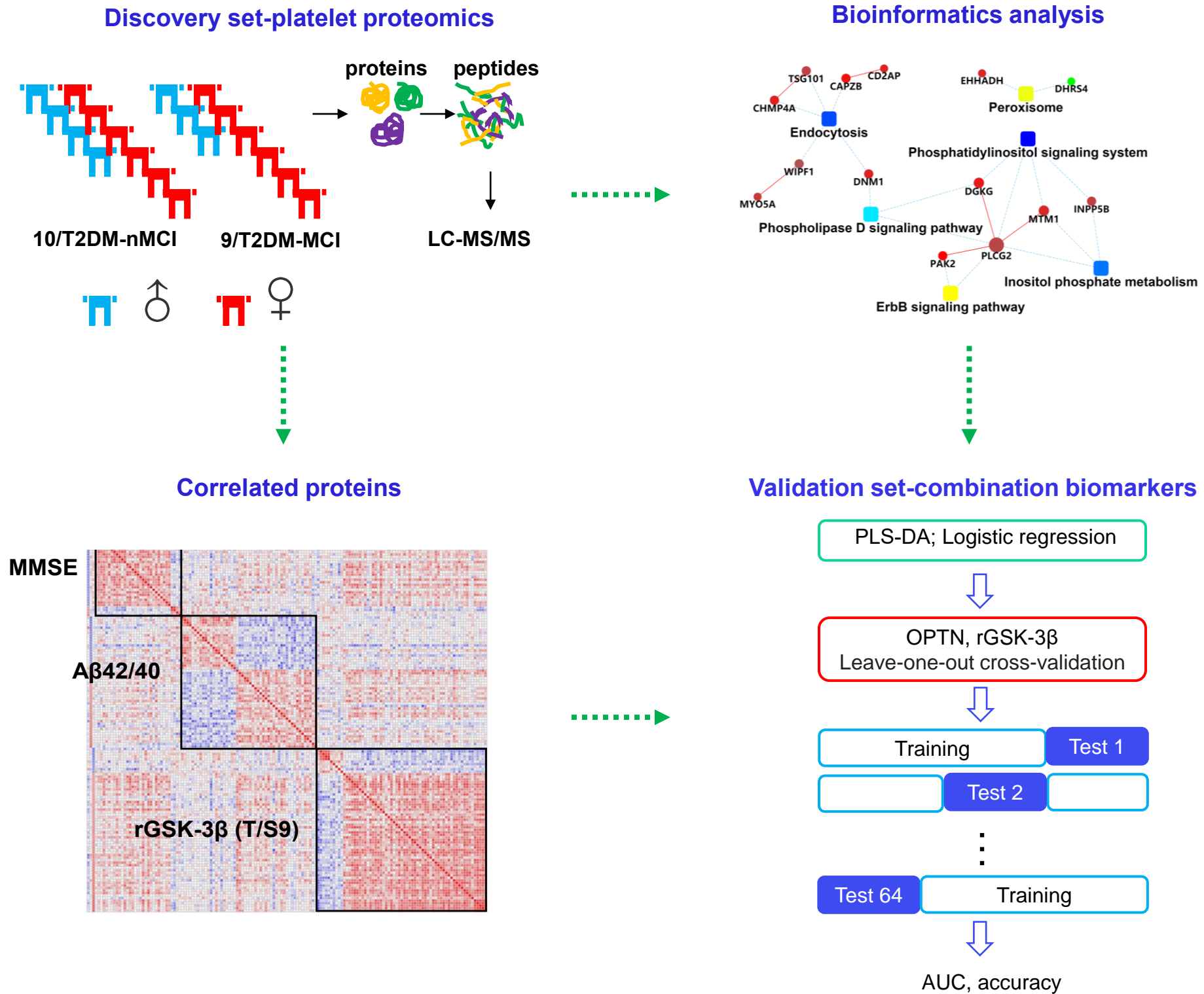

Figure S4

Supplement: Supplementary file 2 — Fig S4 [file ACEL-20-e13469-s003.pdf]
