# Supplementary material for: Platelet biomarkers identifying mild cognitive impairment in type 2 diabetes patients
Source: Aging Cell. 2021 Sep 16;20(10):e13469. doi: 10.1111/acel.13469 (PMC8520722; doi:10.1111/acel.13469)
Supplement: Supplementary file 8 — Table S1 [file ACEL-20-e13469-s001.docx]

| Variable | B | S.E. | Wald | Sig. | Exp(B) | 95 % CI | |
| --- | --- | --- | --- | --- | --- | --- | --- |
|  |  |  |  |  |  | Lower | Upper |
| Olfactory score | 0.507 | 0.331 | 2.347 | 0.126 | 1.660 | 0.868 | 3.176 |
| OPTN | 2.276 | 0.793 | 8.229 | 0.004 | 9.734 | 2.056 | 46.088 |
| rGSK-3β | 3.168 | 1.128 | 7.889 | 0.005 | 23.747 | 2.604 | 216.531 |
| GSK-3β-Ser9 | 0.137 | 1.072 | 0.016 | 0.899 | 1.146 | 0.140 | 9.365 |

B = the estimated logit coefficient. S.E. = the standard error of the coefficient. Wald = B/S.E. Sig. = the significance level of the coefficient. Exp(B) = the odds ratio of the individual coefficient. CI = confidence interval. rGSK-3β = total GSK-3β/Ser9-GSK-3β.
